# Supplementary material for: A cross-sectional investigation of back pain beliefs and fear in physiotherapy and sport undergraduate students
Source: PLoS One. 2023 Apr 20;18(4):e0284754. doi: 10.1371/journal.pone.0284754 (PMC10118113; doi:10.1371/journal.pone.0284754)
Supplement: Attachment — Submitted filename: Review comments on Manuscript Number.doc [file pone.0284754.s003.doc]

**Review comments on Manuscript Number: PONE-D-22-34229. Entitled "Back pain beliefs and fear in physiotherapy and sport undergraduate students"**

Overall, the idea of research is very interesting to be studied nowadays and paper is coherently developed. However, there are some comments and suggestions.

**Abstract**

- Well structured
- Keywords: write it in alphabetical order

**Introduction**

- The abbreviations already mentioned in the abstract so no need to re-write complete terms

**Materials and methods**

- Well structured

**Statistical analysis**

- Well structured

**References**

Update some references is required
