# Supplementary material for: A cross-sectional investigation of back pain beliefs and fear in physiotherapy and sport undergraduate students
Source: PLoS One. 2023 Apr 20;18(4):e0284754. doi: 10.1371/journal.pone.0284754 (PMC10118113; doi:10.1371/journal.pone.0284754)
Supplement: Attachment — Submitted filename: ReplyReview_R1_v1.docx [file pone.0284754.s004.docx]

# Comments are in bold, responses in normal typeset, and *excerpts from the manuscript are in italics.*

# Reviewer #1

## Review comments on Manuscript Number: PONE-D-22-34229. Entitled "Back pain beliefs and fear in physiotherapy and sport undergraduate students". Overall, the idea of research is very interesting to be studied nowadays and paper is coherently developed. However, there are some comments and suggestions. Abstract - Well structured

Reply: We thank the Reviewer for the positive comments. We will address all feedback and suggestions provided by the Reviewer.

## - Keywords: write it in alphabetical order

Reply: We have reordered it to alphabetical order.

## Introduction - The abbreviations already mentioned in the abstract so no need to re-write complete terms

Reply: We thank the Reviewer for this comment. We kindly have to disagree with this feedback, as we feel that similar to Figures and Tables, the main manuscript should be worded independently from the Abstract. We have thus retained the definition of the abbreviations in the main manuscript. However, we will seek the guidance of the Editor on this matter too, during the resubmission process.

## Materials and methods - Well structured Statistical analysis - Well structured

Reply: We thank the Reviewer for the positive comments.

## References Update some references is required

Reply: We thank the Reviewer for this comment. We are unsure if the Reviewer is suggesting we ensure our References are follow PLoS’s guidance, or if new references are needed. We have ensured that our Reference format follows PLoS. We believe that we have tried to cite the latest evidence where available. We are very open to amending any references that the Reviewer thinks needs editing. We hope that the Reviewer can provide the relevant citations for us to consider including.

# Reviewer #2

## The manuscript title should include the type of the study.

# Reply: We have modified the title to read as “A cross-sectional investigation of back pain beliefs and fear in physiotherapy and sport undergraduate students”.

## Conclusion of the study doesn't declare the end result of the study obviously, I think it should be re-written.

Reply: We have added a sentence at the start of the Conclusion, that reads as:

The effect of different undergraduate health and sport study programs on back pain beliefs and fear depended on the study year.

## The author should declare participants inclusion criteria rather than study programmes only.

Reply: We thank the Reviewer for this comment. We followed the broad inclusion criteria of prior similar publications (Inman and Ellard, 2022; Leysen et al., 2021), where the priminary inclusion criteria was that students are enrolled into the program. Given that the aim of the present study was to investigate the beliefs and fear in a representative sample of students in our defined study programs, there was no further inclusion criteria.

# References

1.Inman, J.G.K., Ellard, D.R., 2022. What influences graduate medical students’ beliefs of lower back pain? A mixed methods cross sectional study. BMC Med Educ 22, 633.

2.Leysen, M., Nijs, J., Van Wilgen, P., Demoulin, C., Dankaerts, W., Danneels, L., . . . Roussel, N., 2021. Attitudes and beliefs on low back pain in physical therapy education: A cross-sectional study. Braz J Phys Ther 25, 319-28.
